# Effect of novel Matrix Rhythm Therapy (MaRhyThe®) on neuropathic pain and maximum plantar pressure distribution among type 2 diabetes mellitus patients with peripheral neuropathy

**DOI:** 10.1007/s40200-023-01210-8

**Published:** 2023-04-19

**Authors:** G Arun Maiya, Radhika Aditya Jadhav, Anupama Harihar, Shubha Gundmi, Amratha G. Shetty, Hrishikesh Yadav K, Ozlem Hammond, Ulrich G Randoll, Anil Deshpande, Shreemathi Mayya, Varun C Naik

**Affiliations:** 1 Centre for Diabetic Foot Care and Research, Department of Physiotherapy, Manipal College of Health Profession, Manipal Academy of Higher Education, Madhav Nagar, Manipal, Karnataka 576104 India; 2 Matrix Rhythm Therapy Center, 5 Beswicks Road, Northwich, CW81AP UK; 3 Randoll Institut, Nonprofit Organization for Matrix Research and Education, Lortizingstraβe, Munich, 2681241 Germany; 4 International Medical Technologies, Mumbai, India; 5 Department of Data Science, Prasanna School of Public Health, Manipal Academy of Higher Education, Manipal, 576 104 India; 6 Department of Cardiovascular and Pulmonary Physiotherapy, KAHER Institute of Physiotherapy, Belgavi, India

**Keywords:** Matrix Rhythm Therapy, Extracellular matrix (ECM), Diabetic peripheral neuropathy, Type 2 diabetes mellitus, Plantar pressure

## Abstract

**Background:**

There is a need for a non-pharmacological approach to reduce pain and plantar pressure in diabetic peripheral neuropathy (DPN). Matrix Rhythm Therapy (MaRhyThe®) is a therapeutic modality that works on the principle of physiologic rhythmic oscillations of the body cells. This study aimed to evaluate the effect of MaRhyThe® on neuropathic pain and maximum plantar pressure distribution among type 2 diabetes mellitus patients with peripheral neuropathy.

**Materials and methods:**

A total of 33 participants with DPN were recruited for the study based on inclusion criteria. Maximum plantar pressure was recorded using Win-Track 11K005, and the pain score was obtained using a visual analogue scale. Ten sessions of MaRhyThe® were given to all the participants. Outcome measures were evaluated at the baseline and after 10th session. Paired t-test was performed to analyze the changes in outcome measures.

**Results:**

Participants of DPN were recruited with the average age of 64 ± 9 years, and an average duration of diabetes was 14 ± 9 years were included. Results of the present study found significant improvement in neuropathic pain and plantar pressure in post intervention assessment. (p < 0.05)

**Conclusion:**

In the present study, we found that MaRhyThe® is effective in reducing neuropathic pain and maximum plantar pressure in type 2 diabetes mellitus with peripheral neuropathy.

## Introduction

Diabetes mellitus is a metabolic syndrome with multiple etiology in which there will be a disturbance in the insulin secretion or insulin action or both; that results in chronic hyperglycaemia due to defects in carbohydrate, protein and fat metabolism [1]. Diabetes is present with characteristic signs and symptoms. But the long-time effect of diabetes includes damage and dysfunction of many organs, leading to some significant complications. Diabetic peripheral neuropathy (DPN) is considered one of the most common complications of type 2 diabetes mellitus [2]. According to our clinical observations, we hypothesize that it could also be a sugar utilization disorder because of blockages of flow in the Extracellular Matrix (ECM) in the transit area between capillaries and parenchymal cells. The prevalence of DPN is changing from 3 to 32% across the globe. In south India, the prevalence was 19.1% in the year 2002 [3]. Community-based survey in rural Udupi district has shown a 30.2% of prevalence of sensory neuropathy in 2018 [4]. One among six individuals with diabetes mellitus will be suffering from DPN [5].

DPN may be asymptomatic. But its most peculiar characteristic is pain and paraesthesia [6]. Long standing peripheral neuropathy is also responsible for hamping the quality of life in individuals with type 2 diabetes mellitus [7]. Presence of DPN increases the risk of amputation by two folds. In the presence of simultaneous foot or toe deformity, this risk increases up to 12 times, and among those with a history of ulcers, it is high as 36 fold [4].

Long-term neuropathy and vascular changes due to peripheral vascular disease in DM cause reduced joint mobility, structural changes in the foot, and weakness in the intrinsic muscles of the foot lead to muscular atrophy. These factors result in abnormal joint loading and altered plantar pressure distribution both in standing and during the various parts of the gait cycle. In addition, displacement of the fat pads or decrease in their thickness which otherwise protects and acts as a shock absorption, leads to increased pressure at the metatarsophalangeal joint. This results in peak plantar pressure, microtrauma, and ulcer development in the area [8].

Previous literature has highlighted the effect of both pharmacological and non-pharmacological agents in the prevention and treatment of DPN [8,9]. Currently, very limited treatment options act directly on the natural course of DPN except for rigorous glycemic control. The pain-modulating medications such as nortryptiline, gabapentine, and anticonvulsants can provide symptomatic relief [10]. It is known that artificial insulin is causing chronic inflammation by immune reactions with resulting fibrotic tissue and further degenerative processes. Hence, a perfect alternative might be using specific physical therapies regulating already on an extracellular level with following movement and sports training instead of giving artificial insulin to control the sugar household. Physiotherapy assessment and management include educating the patient on diabetic foot care and screening the patients annually. Monochromatic near-infrared energy and laser therapy can also be used to alleviate the symptoms of neuropathy [4,11,12]. However, there is a need for novel therapeutic approaches with no added side effects.

Following the ideas of systems biology and the studies of the interaction of time space patterns in living systems, a physical therapy device called Matrixmobil was constructed at the University of Erlangen/Nuremberg in the 1990s. The target was to reactivate and synchronize the human body with its corresponding processes on the level of cellular units surrounded by its extracellular matrix. It works on the principle of rhythmic oscillations of the body cells [13,14]. It helps to symmetrically improve circulation and lymphatic flow in the treated area. It also helps regulate circadian rhythm and bring the body’s cells ‘in harmony’ [15]. The various studies on MaRhyThe® have proved its effect on pain and improved functionality in different musculoskeletal conditions [16–18]. But to our knowledge the effect of MaRhyThe® on neurovascular functions and plantar pressure in DPN has not been studied so far. This study aimed to evaluate the effect of MaRhyThe® on neuropathic pain and maximum plantar pressure distribution among type 2 diabetes mellitus patients with peripheral neuropathy.

## Materials and methods

### Trial design

This clinical trial was initiated after approval from the institutional research committee and institutional ethics committee (IEC 171/2020) of Kasturba Hospital, Manipal, India. The trial was registered under CTRI (CTRI/2020/06/025845).

### Participants

A total of 43 patients were screened, of which 33 were recruited for the study at the Centre for Diabetic Foot Care and Research at Kasturba Hospital. Inclusion criteria were: (1) diagnosis of diabetic peripheral neuropathy, (2) history of neurological conditions, (3) musculoskeletal complications. Neuropathy due to other causes, lower limb lymphedema, cellulitis and reduced ankle-brachial index were excluded. Study procedures were explained in detail to the participants who were willing to take part in the study. Written informed consent was obtained from the participants who agreed to take part in the study.

### Intervention

**Fig. 1:**
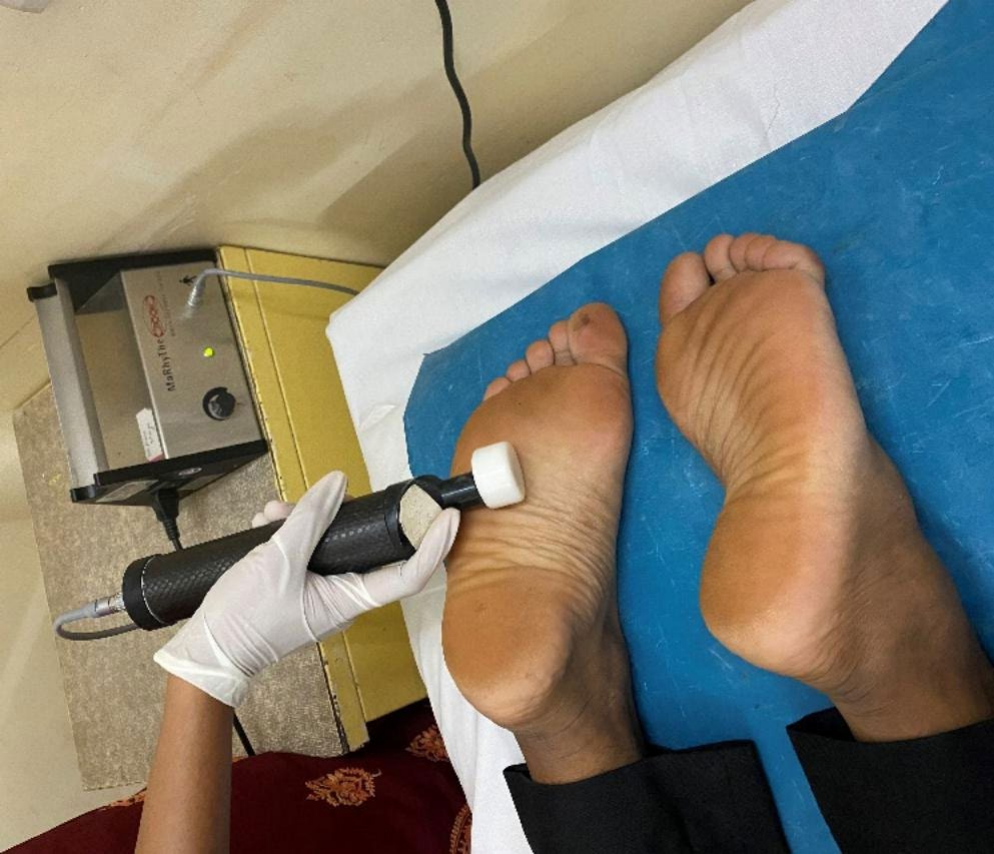
Application of MaRhyThe® on plantar aspect of the foot

The participants were given MaRhyThe® using Matrixmobil® for 10 sessions (Fig 1). Two sessions per week, and each treatment session lasted for one hour. In a prone position, the patient laid down with one pillow under the shin and other under the forehead. The MaRhyThe® was given to the paraspinal area, followed by the calf and plantar aspect of the foot bilaterally. Then in a supine position, the pillow was placed under knees and one under the neck and the treatment was given on the anterolateral aspects of the lower leg and dorsal surface of the foot. During and after the MaRhyThe® application, any discomfort and adverse reactions were recorded.

### Outcomes

Primary outcome measures were neuropathic pain, VPT and maximum plantar pressure. We also evaluated protective sensations, Ankle Brachial Index (ABI), average plantar pressure and foot contact area. All the outcome measures were assessed at baseline and re-assessed after the 10th session and after one month. Observatory findings such as skin changes, toe and foot deformities, and presence of ulcer or infections were also noted. Baseline demographics like age, duration of the DM, other comorbidities, history of previous surgeries were recorded. Participants’ height, weight, fasting blood glucose levels, glycosylated hemoglobin was measured.

#### Diabetic peripheral neuropathic pain evaluation

The neuropathic pain was assessed by using the visual analogue scale (VAS).

#### Vibration perception threshold evaluation

**Fig. 2:**
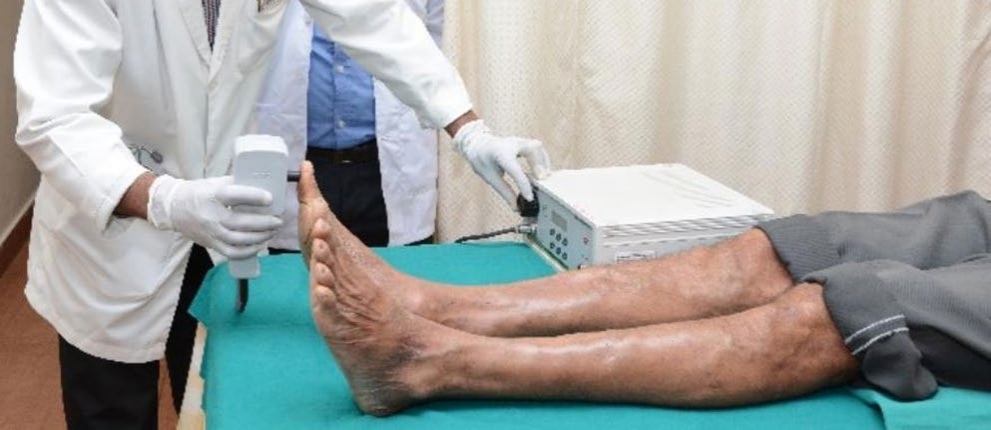
Evaluation of vibration sensation using vibration perception threshold

The vibration sensation was assessed using vibration perception threshold equipment (KODYS BIOTHEZI-VPT) (Fig 2). The test was performed on the forearm or hand to familiarize the test. Then vibration perception was assessed on the ball of the great toe and participant was asked to report the slightest vibration they felt. Participants with a VPT value of > 15 V were included in the study.

#### Plantar pressure analysis

**Fig. 3:**
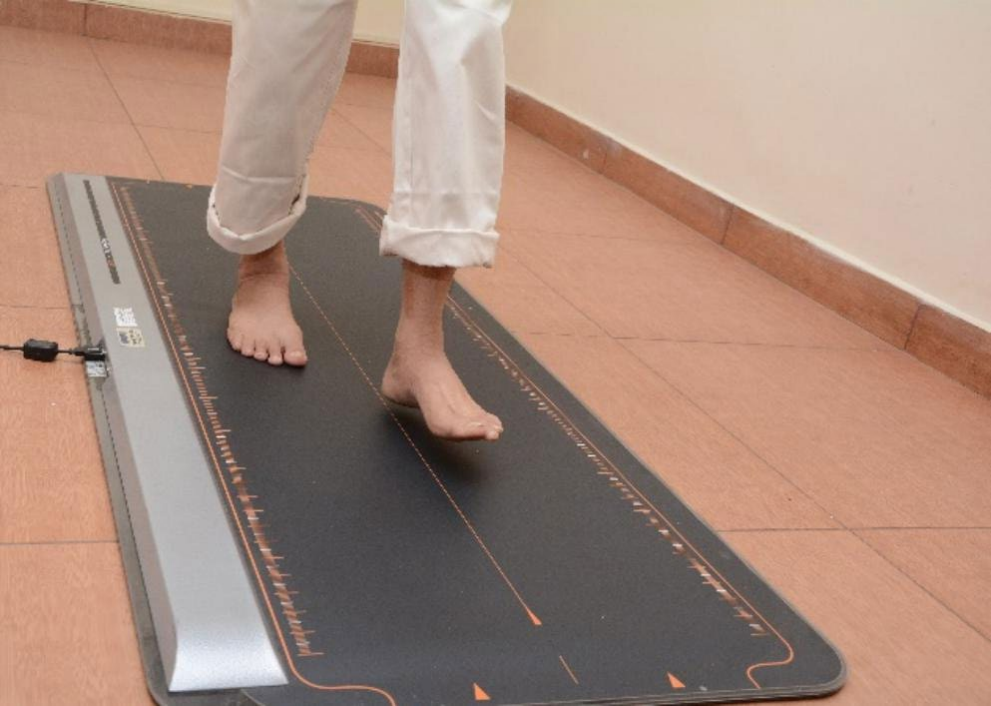
Plantar pressure evaluation using pressure mat

Static and dynamic plantar pressure was measured using Win-Track 11K005 [19]. Participants were made to stand on the platform with a regular base of support for 30 s and were made to walk to evaluate the static and dynamic plantar pressure respectively (Fig 3).

#### Evaluation of protective sensations

**Fig. 4:**
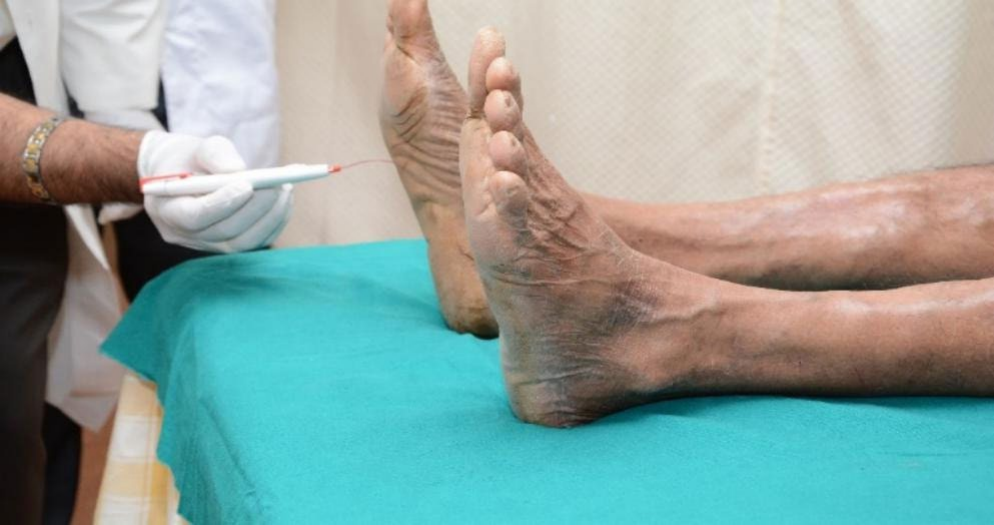
Evaluation of protective sensation using monofilament

The protective sensation of the foot was measured using a 10gram monofilament (Diabetic Foot Care India Pvt Limited) (Fig 4). A standard 6-point method of evaluation was used. First, the procedure was explained to the participant and monofilament was touched to the patient’s arm or hand as to what to expect during the test. A participant was asked to close their eyes and when they felt the monofilament touch, they responded with “yes.“ Monofilament was held perpendicular to the foot and touched to the skin till the monofilament bends at least 1 cm. At each point, the monofilament was held for 2 s. The monofilament test was repeated up to 3 times to confirm if the participant was not able to feel the monofilament at any given point.

#### Ankle-brachial index (ABI) evaluation

The ankle-brachial index was measured using a non-invasive Doppler (smart drop 30EX). Participants were asked to relax for 5 min, seated on a comfortable couch. The cuff was tied 5 cm above the medial malleolus for the lower limb and one inch above the cubital fossa for the upper limb. The probe was placed on the dorsalis pedes, posterior tibial and radial artery to calculate the ankle-brachial index.

### Sample size

Sample size was calculated by using the formula, *n =* *2 (Z*_*1- α/2*_*+ Z*_*1-β*_*) S2/ d*^*2*^; considering the findings from the pilot study; S = 11.1 and d = 5. The sample size calculated was 28 (without attrition). Considering 20% attrition we decided to recruit 33 participants in the study.

### Statistical methods

SPSS version 16 was used to analyze the data. A descriptive statistic test was done to analyze the demographic data. Paired t-test was used to analyze the pre-post changes in the outcome measure.

## Results

A total of 43 participants were screened and 33 participants were recruited. The average age of the participant was 64 years (41–80 years) and the average duration of the diabetes was 14 years (1–35 years). The detailed baseline demographics of the participants is given in Table 1.

**Table 1:** Demographic characteristics of participants

*N = 33*	Mean ± SD
Age (years)	64.8 ± 9.6
Height (Cm)	165.3 ± 8.2
Weight (Kg)	69.9 ± 10.7
Diabetes duration (years)	14.03 ± 9.1

All the outcome measures were taken at baseline and after the 10th session of the treatment. There was a statistically significant improvement in pain (MD = 3.182 ± 1.402) and VPT (MD = 7.5803 ± 4.94) (Table 2).

**Table 2:** **Effect of MaRhyThe® on neuropathic pain and VPT**

*N = 33*	*Paired Differences*			*Sig. (2-tailed*)
	*Mean ± SD*	*95% Confidence Interval of the Difference*		
		*Lower*	*Upper*	
Baseline VAS	5.76 ± 1.58			
Post VAS	2.58 ± 1.41			
Pre-VAS - Post VAS	3.182 ± 1.4	2.685	3.679	0.000
Baseline VPT	32.39 ± 12.98			
Post VPT	24.81 ± 11.66			
VPT-PRE - VPT-POST	7.5803 ± 4.94	5.8284	9.3322	0.000

*VAS: Visual Analogue Scale, VPT: Vibration Perception Threshold*

**Table 3:** Effect of MaRhyThe® on static plantar pressure (average plantar pressure, maximum plantar pressure, contact area)

*N = 33*	*Paired Differences*			*Sig. (2-tailed)*
	Mean ± SD	***95% Confidence Interval of the Difference***		
		***Lower***	***Upper***	
Baseline average pressure	119.221 ± 35.0			
Post average pressure	107.228 ± 22.55			
AP-PRE - AP-POST	12.0136 ± 32.37	0.5332	23.4941	0.041
Baseline maximum pressure	267.774 ± 60.16			
Post maximum pressure	242.327 ± 40.86			
MP-PRE - MP-POST	25.4470 ± 58.68	4.6366	46.2574	0.018
Baseline contact area	32.52 ± 6.29			
Post contact area	35.52 ± 5.89			
contact area-pre - contact area post	-3.000 ± 5.87	-5.083	− 0.917	0.006

Table 3 indicates the changes in plantar pressure parameters (average plantar pressure, maximum plantar pressure and contact area). Results of the present study found significant improvement in neuropathic pain and plantar pressure in post-intervention assessment. We found a significant improvement in all the parameters of plantar pressure. (p < 0.05).

There was a significant improvement in the VAS score after the 10th session of the treatment. On the visual analogue scale, the difference in the baseline and post-intervention was 3 units. This indicated a 30% reduction in pain due to MaRhyThe®. There was a marginal change in the ABI, which was clinically significant but could not reach the level of statistical significance. Along with the improvement in the pain score, there was also an improvement in the neuropathic profiles, that is, VPT. We found a 15% improvement in VPT among the participants. We also found a 22% improvement in the protective sensations, which was assessed by using 10 gm monofilament. There was a significant improvement in the static plantar pressure parameters. We also found that the improvement in outcome measures was maintained at one month follow-up assessments.

## Discussion

In this pre-post measurement study, we evaluated the effect of novel MaRhyThe® on neuropathic pain and maximum plantar pressure distribution among 33 type 2 diabetes mellitus patients with peripheral neuropathy. Each participant received 10 sessions of MaRhyThe®.

### Effect of MaRhyThe® on neuropathic pain

This study found a significant reduction in neuropathic pain due to MaRhyThe®. Neuropathic pain is pain arising due to a lesion affecting the somatosensory system [20]. Around 20% of diabetic patient experiences neuropathic pain, and the incidence is rising. The pharmacological management of neuropathic pain is a complex task. Current international guidelines revealed that only 30% of patients experience a reduction in pain with pharmacological management up to 30% [21. MaRhyThe® by improving microcirculation and triggering the metabolism [15]. This might be the possible reason for finding a reduction in pain.

### Effect of MaRhyThe® on static plantar pressure

The study found a significant improvement in neuropathic profile and plantar pressure. Alteration in the mechanism of the lower limb and undetected trauma is responsible for increased plantar pressure in DPN [22]. Elevated plantar pressure is a potential site of ulceration. Various factors influence the plantar pressure distribution. These include age, glucose levels at the time of measurement, muscle tightness in the lower limb, foot arches and balance [23]. MaRhyThe® regulates the extracellular matrix back to normal so that derailed extracellular and cellular processes can start again, leading to self-organization/self-healing. This approach is postulated to be beneficial in cases of diabetic neuropathy as it accelerates structural and functional nerve regeneration and relaxes muscle fibers [14].

### Effect of MaRhyThe^®^ on neuro-vascular function

The mechanism of MaRhyThe® would have triggered the metabolism by improved microcirculation, which ABI often underestimates. In our study, we found a marginal change in the ABI in 10 sessions. Previous studies have shown that MaRhyThe® improves blood circulation by up to 35% in normal individuals [15] and in some studies which included mechanical vibrations blood circulation improved from 20% to up to 46% [24]. Near the body’s cells, the vibration provides a pumping action that helps to transport nutrients, defense substances and waste products to and from the body. If the muscle processes no longer function properly, the cell oscillations slow down to the point of congestion, and the affected cell areas are no longer adequately supplied. Studies suggest that MaRhyThe® improves microcirculation more than conventional massage does.

The compression effect created by the application of MaRhyThe® can cause more soft tissue mobilization and more afferent stimuli by the vibration effect. The vibration frequency created by the MaRhyThe® device is thought to be compatible with the natural vibration frequency of the muscle, which is considered to contribute to the therapeutic effectiveness of MaRhyThe® [19].

In the present study, a heterogeneous population with various comorbidities along with type 2 diabetes mellitus was included. The participants with a long-standing duration of diabetes were also included. The average duration of diabetes was 14 years. Out of the total, 23 participants had a duration of diabetes of more than 10 years. This is one of the reasons for marginal improvement in the parameters. We also found that the improvement in outcome measures was maintained in one month post follow-up. Moreover, reducing the symptoms would help in a better quality of life.

### Strength and limitation

To the best of our knowledge, this is the first study that reported the effect of MaRhyThe® on neuropathic pain and plantar pressure among diabetic peripheral neuropathy. The limitation of the study includes a lack of long-term follow-up.

### Future recommendation

The studies can include changes in the inflammatory biomarkers with long term follow-up.

## Conclusion

In the present study, we found that MaRhyThe® is effective in reducing neuropathic pain and maximum plantar pressure in type 2 diabetes mellitus with peripheral neuropathy.
